# Broadband and Wide Field-of-View Refractive and Meta-Optics Hybrid Imaging System for Mid-Wave Infrared

**DOI:** 10.3390/nano15070566

**Published:** 2025-04-07

**Authors:** Bo Liu, Yunqiang Zhang, Zhu Li, Bingyan Wei, Xuetao Gan, Xin Xie

**Affiliations:** 1 Key Laboratory of Light Field Manipulation and Information Acquisition, Ministry of Industry and Information Technology, and Shaanxi Key Laboratory of Optical Information Technology, School of Physical Science and Technology, Northwestern Polytechnical University, Xi’an 710129, China; 2 National Key Laboratory of Air-Based Information Perception and Fusion, Luoyang 471000, China; 3 Institute of Machinery Manufacturing Technology, China Academy of Engineering Physics, Mianyang 621900, China

**Keywords:** hybrid imaging system, metasurfaces, wide field-of-view, mid-wave infrared

## Abstract

We propose a wide field-of-view (FOV) refractive and meta-optics hybrid imaging system designed for the mid-wave infrared spectrum (3–5 μm) to address the challenge of high-quality imaging in wide FOV applications. The system consists of only three refractive lenses and two metasurfaces (one functioning as a circular polarizer and the other as a phase element), with a total length of 29 mm. Through a detailed analysis of modulation transfer function curves and spot diagrams, the system achieves 178° FOV while maintaining exceptional imaging performance across a temperature range of −40 °C to 60 °C. The system demonstrates the potential for extending applications to other wavelengths and scenarios, thereby contributing to the advancement of high-performance compact optical systems.

## 1. Introduction

Wide field-of-view (FOV) optical imaging systems in the mid-wave infrared (MWIR) spectrum are crucial in various fields, including environmental monitoring, autonomous driving, medical imaging, and aerospace. Early optical designs primarily utilized fisheye and panoramic lenses, achieving a wide FOV by selecting suitable lens parameters or employing specific configurations [1,2,3]. However, these systems frequently suffer from substantial aberrations, chromatic distortions, and image artifacts that prevent the realization of high-quality imaging. Researchers have introduced aspherical and diffractive optical elements to mitigate these issues, significantly reducing aberrations and enhancing imaging clarity and uniformity [4,5,6,7]. Aspherical lenses provide a more consistent image plane over an expanded FOV, while diffractive optics leverage specially designed optical structures to broaden the range of applications further. Additionally, microlens array systems effectively achieve a wide FOV imaging by integrating numerous small lenses, facilitating three-dimensional imaging and depth information acquisition [8,9,10,11]. Nevertheless, these systems encounter several challenges, including increased optical path complexity and packaging difficulties due to the spacing between micro-lenses.

Metasurfaces are artificially engineered architectures that can manipulate multiple optical degrees of freedom at subwavelength scales, allowing for highly flexible and efficient control of light propagation and reflection [12,13,14,15]. Although conventional monolithic metalenses have demonstrated remarkable potential for wide-field imaging applications [16,17,18,19], the necessity for broadband phase compensation and off-axis aberration optimization on a single surface forces them to trade operational bandwidth for expanded FOV, resulting in inherent limitations in concurrently achieving both broad bandwidth and wide FOV [20,21]. While deep learning-enhanced design methodologies can improve metalens performance in FOV and bandwidth, these methods substantially escalate design complexity and exhibit heavy reliance on the effectiveness of algorithmic training [22].

In this work, we propose a refractive and meta-optics hybrid imaging system, which integrates refractive lenses with metasurfaces to balance FOV and bandwidth requirements. By offloading FOV extension tasks to refractive elements, the metasurface design complexity is significantly reduced, while enabling simultaneous achievement of an ultra-wide FOV and broadband operation. Furthermore, careful selection of lens materials provides compensation for thermal focal shifts, thereby enhancing the overall imaging performance of the system. Recent advancements in hybrid optical systems, as demonstrated in applications such as augmented reality (AR) displays [23] and advanced optical architectures [24,25,26], have achieved significant improvements over conventional designs. These systems exhibit reduced size and weight, enhanced wavelength and FOV controllability, and greater design flexibility while maintaining excellent optical performance.

Our hybrid optical system features a compact total length of 29 mm, comprising three refractive lenses and two metasurfaces (one serving as a circular polarizer and the other as a phase element). This configuration enables wide-angle imaging with a FOV approaching 180° in the MWIR spectrum (3–5 μm). An athermal design was achieved by selecting appropriate lens materials to ensure the performance of the system under extreme conditions within the temperature range from −40 °C to 60 °C. Further analysis of the modulation transfer function (MTF) plots and spot diagrams at different temperatures demonstrated that the MTF values corresponding to the cutoff frequencies exceeded 0.52, with the root mean square (RMS) radius being smaller than the Airy radius and the geometric (GEO) radius being smaller than the pixel size. The results showed that the system achieved a 178° FOV within the MWIR spectrum over the temperature range from −40 °C to 60 °C. To further reduce the system size and improve the system integration, a circular polarizer was designed based on a bilayer metasurface whose front and back layers are equivalent to a linear polarizer and a quarter-wave plate. Meanwhile, a metasurface phase element was designed to match the phase distribution of the binary 2 surface, with the matching error being less than 3.5 degrees. In addition, the designed imaging system has significant advantages in terms of FOV and system size compared with commercial cameras. This design illustrates the feasibility of maintaining excellent imaging quality while reducing system complexity.

## 2. Discussions and Results

### 2.1. Design and Analysis of the Optical Imaging System

**Figure 1:**
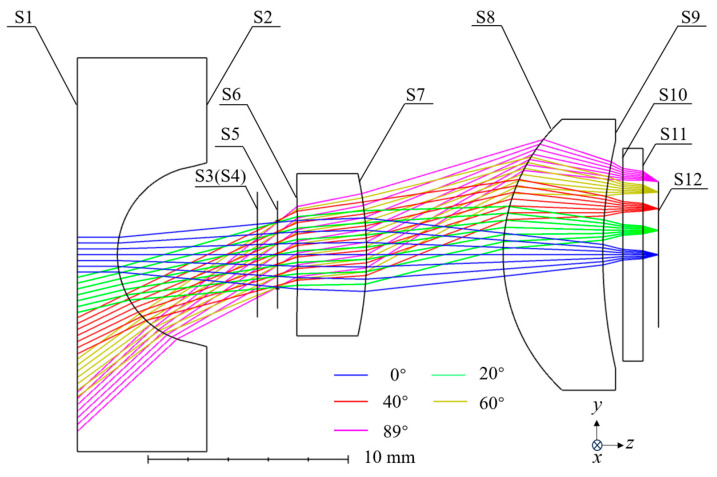
Schematic diagram of the system structure.

The design parameters of the system are presented in Table 1. The system employs three refractive lenses and two metasurfaces that serve as a circular polarizer and a phase element, achieving an optimal balance between system complexity and imaging performance. The refractive lenses are the primary imaging elements, effectively bending light rays and enhancing imaging precision. A metasurface polarizer is positioned posterior to the first refractive lens to convert incident light into circular polarization states, whereas a metasurface phase element is located at the terminal optical surface to execute wavefront aberration correction. Ansys Zemax OpticStudio 2024 R1 was used to simulate the performance of the optical imaging system, and the final structural layout of the system is shown in Figure 1. The system configuration closely approximates an image-space telecentric design, which helps reduce measurement errors introduced during focus adjustments. A binary 2 surface was used to simulate the required phase distribution of the metasurface phase element, with the phase profile expressed as

(1) $$\varphi =M_{\mathrm{order}}\sum_{i=1}^{N}A_{i}\rho^{2i}$$

where *φ* represents the phase, *M*_order_ denotes the diffraction order, *N* is the number of terms in the polynomial series, *ρ* refers to the normalized radial aperture coordinate, and *A_i_* is the coefficient of the 2*i*-th power of *ρ*. In this case, *M*_order_ is set to 1st order, *N* is 4, and *ρ* is the ratio of *r* to *R*, where *r* is the radius corresponding to different positions on the metasurface phase element. *R* is the normalized radius, with *R* set to 1 mm. As a result, the values of *A*_1_~*A*_4_ are determined to be −24.444293, 1.884176, −0.094382, and 1.575407 × 10^−3^, respectively.

Table 2 provides detailed parameters of the system’s structure. To ensure compatibility with the geometric metasurface, the system incorporates two Jones matrix surfaces as a linear polarizer and a quarter-wave plate, resulting in circularly polarized light incidence. The remaining surfaces are either planar or easily manufacturable spherical surfaces, reducing the system’s complexity. Additionally, the short back focal length and the flat final surface of the system facilitate seamless integration with other components, enhancing the overall system integration level. Furthermore, since temperature variations can alter the radius and refractive index of lenses, thereby inducing thermal focal shift, a combination of lenses fabricated from distinct materials has been strategically employed in the system design to balance thermal focus shift. This material-specific compensation mechanism effectively balances the thermal defocusing effect across operational temperature ranges.

**Figure 2:**
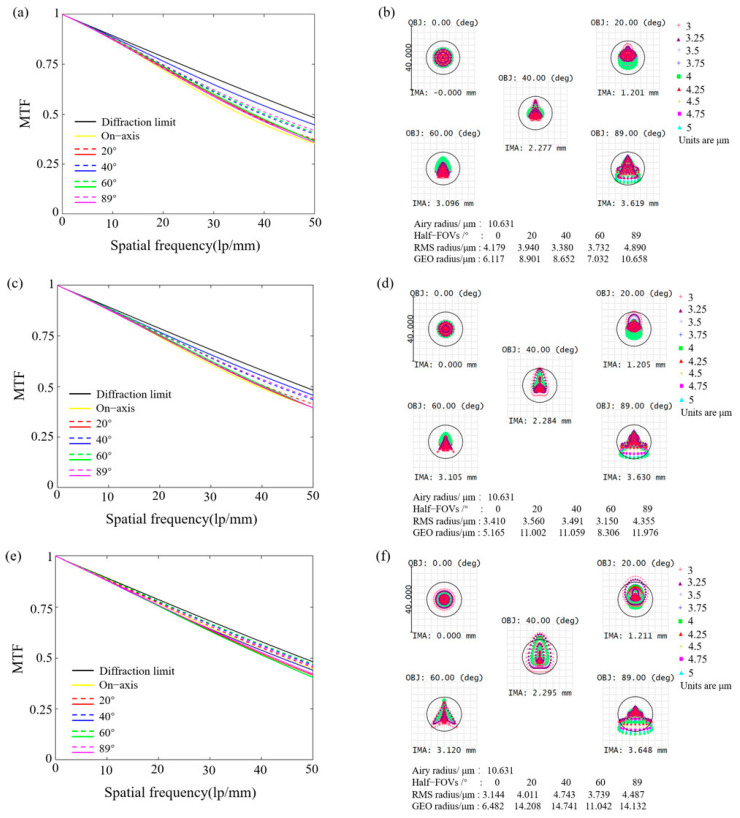
Imaging characterization of the system. (**a**,**c**,**e**) Optical MTF for different half-FOV (dashed curves: tangential MTF; solid curves: sagittal MTF) at operating temperatures of −40 °C, 0 °C, and 60 °C, respectively. (**b**,**d**,**f**) Spot diagram for different half-FOV within the same temperature range.

The MTF and spot diagrams effectively indicate the system’s imaging performance. Consequently, the MTF and spot diagrams at temperatures of −40 °C, 0 °C, and 60 °C were analyzed to validate the operating temperature range of the system, as shown in Figure 2. The final cutoff frequency of the system depends on the minimum value between the cutoff frequency corresponding to the diffraction limit and the cutoff frequency of the detector used. The cutoff frequency of the diffraction-limited system can be obtained by *v*_1_ = 1/(*λ*F/#), where *λ* and F/# represent the central wavelength and f number, respectively. Therefore, the cutoff frequency corresponding to the system’s central wavelength is 125 lp/mm. Additionally, the cutoff frequency of the detector is related to the pixel size, with a value of *v*_2_ = 1/(2*p*), where *p* represents the pixel size. Here, we assume the detector has a pixel size of 15 µm, resulting in a *v*_2_ of 33.3 lp/mm, which is lower than the *v*_1_. Therefore, the system’s cutoff frequency is determined to be 33.3 lp/mm. Figure 2a,c,e present the MTF curves for half-FOV at temperatures of −40 °C, 0 °C, and 60 °C. Since the system is symmetrical about the optical axis, the imaging analysis only presents the results for different half-FOV from 0° to 89°. Despite the slight fluctuations in the MTF curves due to variations in operating temperature, all values remain very close to the diffraction limit, exceeding 0.53 at the cutoff frequency. Furthermore, the analysis of the corresponding spot diagrams indicates that the RMS radius is less than the Airy radius (10.631 µm), and the GEO radius is smaller than the pixel size (15 µm), achieving the detector’s limit resolution, as shown in Figure 2b,d,f.

**Figure 3:**
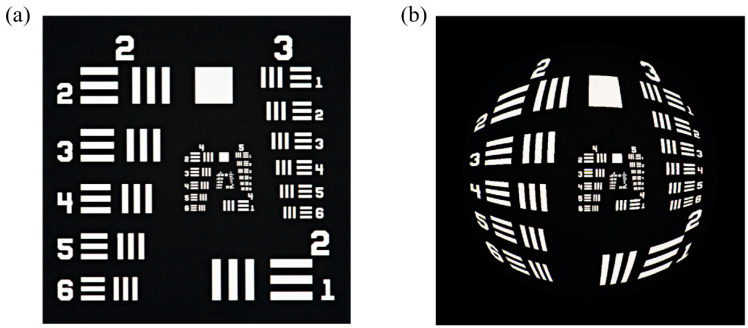
Imaging simulations. (**a**) Ground truth image; (**b**) Simulation result.

To further demonstrate the imaging performance of the designed hybrid system, a 780 × 780 pixels USAF 1951 resolution test chart was employed as the input to analyze the full 178° FOV imaging results, as shown in Figure 3. The comparison between the ground truth image (Figure 3a) and the output image (Figure 3b) reveals exceptional sharpness, well-defined details, and clear contours. Notably, the system’s ultra-wide 178° FOV introduces geometric distortion that progressively intensifies toward the image periphery. However, this distortion does not degrade the intrinsic resolution and can be corrected through post-processing algorithms.

### 2.2. Characterization of the Circular Polarizer and the Geometric Metasurface

**Figure 4:**
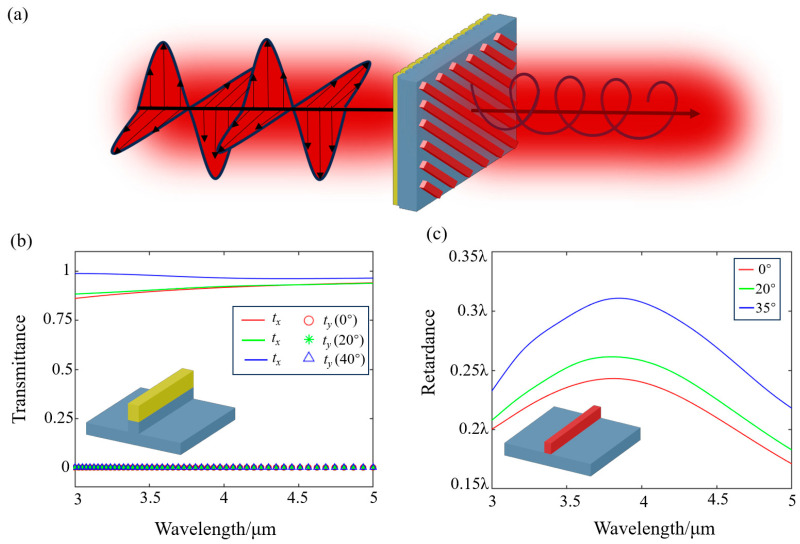
Characterization of circular polarizer. (**a**) A bilayer metasurface achieves conversion of normal light to circular polarization. (**b**) Transmittance of *x*-polarized and *y*-polarized light through the front metasurface under the incidence angles of 0°, 20°, and 40°. Inset shows the cell structure. (**c**) Retardance of the quarter-wave plate under the incidence angles of 0°, 20°, and 35°. Inset shows the unit structure.

To further reduce the system size and enhance its integration level, a circular polarizer is designed using a bilayer metasurface, which is equivalent to a linear polarizer and a quarter-wave plate, as shown in Figure 4a. Figure 4b illustrates the polarization response of the linear polarizer under the incidence angles of 0°, 20°, and 40°, where the transmittance is greater than 0.86 for *x*-polarized light and close to 0 for *y*-polarized light. Furthermore, the average polarization extinction ratio (PER) across the operational band is calculated to be 62.4 dB, 53.5 dB, and 60.1 dB for incidence angles of 0°, 20°, and 40°, respectively. As shown in the inset, a double-layer grating structure is employed to achieve a perfect polarization filtering, the period of the unit structure is 0.35 μm, and the duty ratio is 0.5. The etching depths of Au and MgF_2_ layers are 0.35 μm and 0.25 μm, respectively. Figure 4c characterizes the phase retardation of the designed quarter-wave plate. One can see that the phase retardation between the *x*-polarized and *y*-polarized light maintains approximately 0.25*λ*, with average phase errors of 0.0282*λ*, 0.0131*λ*, and 0.0288*λ* at incident angles of 0°, 20°, and 35°, respectively. The unit structure has a period of 0.792 μm, and the width and height of the silicon strip are 0.521 μm and 0.82 μm, respectively. Notably, due to the refractive optics, rays at the extreme field (89° half-FOV) intercept the metasurface polarizer at a much smaller angle (~34°). This indicates that the designed polarizer can operate effectively within the angular range. Additionally, the designed metasurface polarizer can be fabricated using existing processes such as electron-beam lithography and nanoimprint lithography. For practical implementation, precise alignment of the metasurface element with the optical axis should be ensured to maintain system performance.

**Figure 5:**
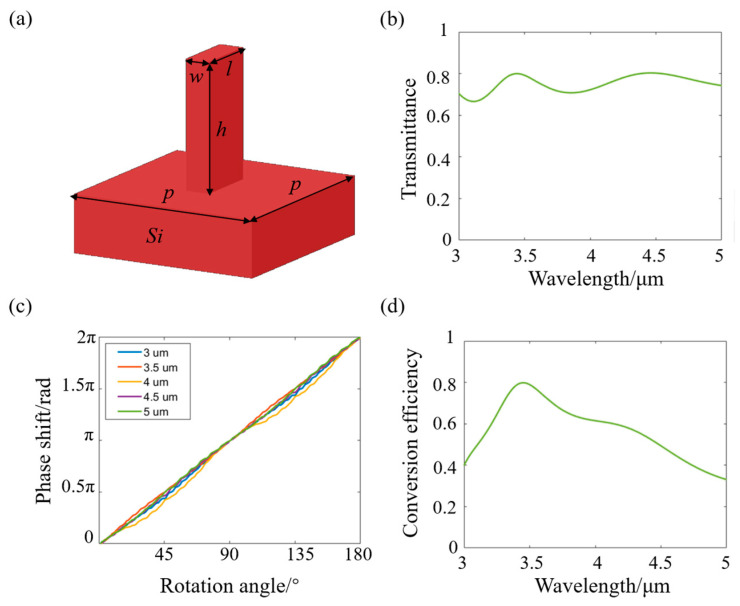
Unit structure and the optical responses. (**a**) Diagram of the unit structure. The period *p* is 1.5 μm, the length l is 1.3 μm, the width w is 0.17 μm, and the height h is 4 μm. (**b**) Simulated transmittance of the silicon pillar in the wavelength range of 3–5 μm. (**c**) Geometric phase characterization. (**d**) Polarization conversion efficiency in the wavelength range of 3–5 μm.

Since the phase shift induced by geometric phase is independent of wavelength, a geometric metasurface was designed to achieve the phase distribution of the binary 2 surface. Silicon materials exhibit good transmittance in the MWIR range and possess excellent thermal conductivity, facilitating the implementation of an athermal design system. Therefore, an all-silicon structure was adopted in the design of the metasurface. To analyze the optical response of the silicon pillars, the Finite-Difference Time-Domain (FDTD) method was employed for simulation and optimization. The optimized parameters of the unit cell structure are illustrated in Figure 5a, featuring a high aspect ratio that enables robust fabrication via the Bosch process [27,28]. The simulated transmittance in the wavelength range of 3–5 μm is shown in Figure 5b, with the average transmittance exceeding 0.72 within the operating wavelength band. Figure 5c illustrates the structure’s response to the geometric phase, clearly showing the relationship of *φ* = 2*σθ*, where *σ* = ±1 denotes left- and right-handed circularly polarized light, indicating that the phase shift is twice the rotation angle [29]. To evaluate the polarization conversion efficiency of the structure, the transmittance of the cross-polarized light was also obtained by simulation, as shown in Figure 5d, which indicates that the average conversion efficiency of the structure is greater than 0.59 in the broadband of 3–5 μm. Notably, while the designed system achieves an ultra-wide 178° FOV, the maximum incidence angle on the metasurface phase element has been confined to a narrow angular regime (~20°) through optical path optimization. This angular constraint effectively mitigates efficiency degradation and phase modulation inaccuracies typically associated with a high incidence angle.

**Figure 6:**
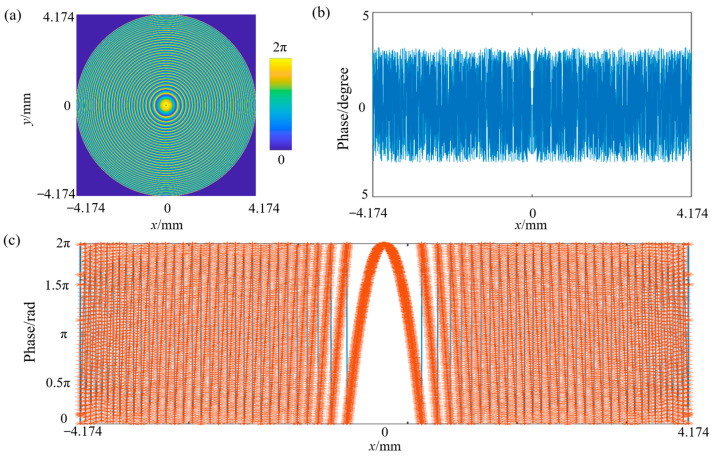
Target phase and phase matching results. (**a**) Phase distribution of the binary 2 surface. (**b**) Phase matching errors of the metasurface. (**c**) Phase matching results of the metasurface. The blue solid line and the red dotted line represent the phase curve of the binary 2 surface and the phase distribution of the metasurface, respectively.

The metasurface is designed based on the phase distribution of the binary 2 surface, as depicted in Figure 6a. Under large-angle incidence, the system experiences significant aberrations and chromatic dispersion, necessitating a steeper phase gradient. As a result, the phase variation becomes increasingly pronounced near the edges. With the benefit of the excellent geometrical phase relationship of the cell structure, the phase shift provided by the cell structure very closely fits the phase of the binary 2 surface with a matching error almost within ±3.5 degrees, as shown in Figure 6b,c.

Table 3 compares the key parameters between common commercial MWIR cameras and the system developed in this work. The results show that the proposed system covers the broadband of 3–5 μm and offers substantial advantages in FOV and total length, indicating significant potential for applications in remote sensing, infrared imaging, and security fields.

## 3. Conclusions

In summary, a broadband and wide FOV refractive and meta-optics hybrid imaging system is proposed to address the key challenges encountered in traditional MWIR imaging systems and metasurfaces. A binary 2 surface was selected to simulate the symmetric phase distribution of the geometric metasurface in the system. Through fine-tuning the parameters of the refractive lenses and optimizing the metasurfaces parameters, a structure composed of three refractive lenses and two metasurfaces (functioning as a circular polarizer and a phase element) was achieved, with a total length of 29 mm. Additionally, an athermal design was achieved by selecting suitable lens materials to ensure performance under extreme conditions. The MTF results and spot diagrams were analyzed between −40 °C and 60 °C, demonstrating values above 0.52 at 33.3 lp/mm, close to the system’s diffraction limit. Furthermore, the RMS radius is smaller than the Airy radius, and the GEO radius is smaller than 15 μm, demonstrating a 178° FOV imaging quality within the temperature range. Meanwhile, a circular polarizer was designed based on a bilayer metasurface to further reduce the system size and improve the system integration. A metasurface was also designed to match the phase distribution of the binary 2 surface, with the matching error being less than 3.5 degrees. Moreover, comparisons with commercial MWIR cameras confirm that the proposed system excels in wide-field imaging. The integration of metasurfaces provides several advantages, including reduced system size, enhanced integration, and improved image quality. This study offers a solution for maintaining broadband imaging over large FOV. Additionally, the introduction of a metasurface expands the system’s potential functionalities, such as polarization and spectral imaging, further broadening its application scenarios.

## Figures and Tables

**Table 1:** Design parameters of the system.

Parameters	Values
Working waveband/µm	3~5
F/#	2
Field of view/°	178
Pixel/µm	15
Effective focal length/mm	3.51
Total length/mm	29.00
Operating temperature/°C	−40~60

**Table 2:** System structural parameters.

Surface Number	Surface Type	R/mm	T/mm	Material
S1	Plane	Infinity	2.000	KCl
S2	Sphere	4.473	7.002	——
S3	Jones Matrix	Infinity	1.000	——
S4	Jones Matrix	Infinity	0	——
S5	Stop	Infinity	1.000	——
S6	Sphere	−141.788	3.448	Si
S7	Sphere	−19.289	6.826	——
S8	Sphere	9.237	4.983	KRS5
S9	Sphere	25.705	1.000	——
S10	Plane	Infinity	1.000	Si
S11	Binary 2	Infinity	0.779	——
S12	Image plane	Infinity	——	——

**Table 3:** The comparison between common MWIR cameras and this system.

Name	Band/µm	FOV/°	Total Length/mm	Operating Temperature/°C
This work	3~5	178	29.00	−40~60
MD4.93F2WR 2	3.5~5	120 × 90	>50	−40~60
Xenics Tigris-640-MCT	3.7~4.8	-	>100	−40~60
FLIR X6981-HS InSb™	3~5	-	>100	−20~50
FLIR A6700 MWIR	3~5	34 × 26	>100	−25~55
Specim FX50	2.7~5.3	60	>100	5~40

## Data Availability

The original contributions presented in this study are included in the article. Further inquiries can be directed to the corresponding author.
